# The role of diclofenack on inducing of aplasia cutis congenita: a case report

**DOI:** 10.1186/1757-1626-2-150

**Published:** 2009-10-12

**Authors:** Laura Pajaziti, Syzana Rexhepi, Ylfete Shatri-Muça, Mybera Ferizi

**Affiliations:** 1 Department of Dermatology, University Clinical Centre of Kosovo, Prishtina, Republic of Kosovo

## Abstract

**Background:**

Aplasia cutis congenita is a disorder where e newborn child is missing skin from certain areas. It is a rare condition with no particular race or sex more at risk. May occur by itself or be associated with other physical syndromes or disorders. A classification system exists for aplasia cutis congenital consisting of 9 groups, based on the number and location of the skin defects and the presence or absence of other malformations. Causes of aplasia congenital could be heredity, teratogenic substances, placental infarcts, intrauterine infections, ectodermal dysplasias etc. Diagnosis is made based on the clinical findings. Prognosis depends of the other organs malfunction level and lesions size.

**Case report:**

Our case was an 22 months old Albanian girl, who was recommended to dermatology for a consultation by a pediatric surgeon because of the changes she had on her parietal part of the scalp with missing hair areas. The child has stenosis congenita ani and to her was installed stoma. In order to investigate other accompanied anomalies of the disease, there are made specific consults by neurologist, orthopedist, cardiologist, nephrologists and citogenetics.

**Conclusion:**

It was found out a minor visual discoordination, Sy Floppy, Digiti V superductus pedis bill. Laxitas articularum generalisata.

It was a great challenge for us to find out that during the first trimester of the pregnancy (unplanned pregnancy), her mother used Diclofenac.

Since there is limited information regarding to teratogenic effects of diclofenac, we considered it interesting to present this case.

## Introduction

Aplasia cutis congenita is one of the disorders of the skin embrional development. It is characterized by a limited defect of the skin where the skin layers and its adnexes are not created. This feature is rare, about 1 in 3000 alive born children [1-3].

Except sporadic cases, there are familiar cases, which are explained by autosomal -dominant heredity [4]. Causes of aplasia could be teratogenic substances, placental infarcts, intrauterine infections, ectodermal displasias etc [5,6].

The disorders during the skin development can include one or all skin layers. Sometimes the other deep structures like fascia, ossa and dura are missing (which can be followed by different cosmetic defects of the skin until the deformities incompatible with life). Aplasia cutis congenita is manifested by the defects of the skin mostly oval, 1-3 cm in diameter, with localization on the parietal part of scalp (60%) and rarely on the face and extremities. It belongs to the group of congenital cicatricial alopecia. Aplasia cutis congenita can be associated by a lot of anomalies as: labium leporinum, polycystic kidneys, psychomotor retardation, developing disorders of the eyes, spinal cord, ears, chromosomal aberationes, cutis marmorata, congenital organoid nevus on the scalp and face, anomalies on extremities, intestines and genital organs. Based on the presence of these anomalies and their association with the other diseases as bullous epidermolysis, growth and development stagnation, etc, congenital aplasias of the skin are classified in 9 groups.

Diagnosis is made based on the clinical findings, and the histopathology is not necessary.

Prognosis depends of the other organs malfunction level and lesions size.

The lesions can resolve spontaneously, but sometimes is needed surgery operation (punch-graft-transplant).

## Case presentation

Patient aged 22 months, Albanian female, hospitalized in Clinic, by pediatric surgeon recommendation, because of the changes on the scalp with missing hair areas. This alteration persists from the birth without tendency for shape changing.

**Figure 1:**
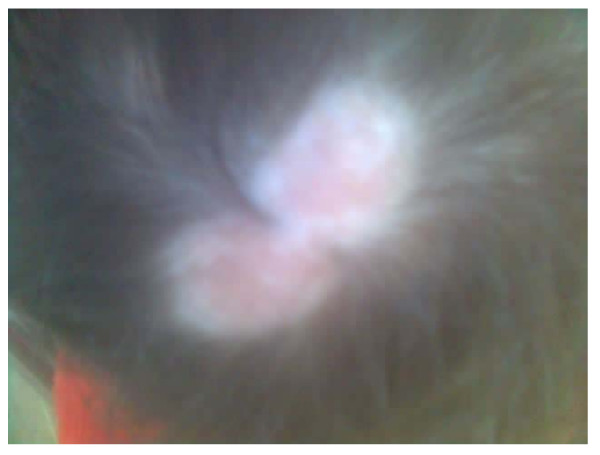
**The parietal part of the scalp appear two oval shaped areas without hair, confluated, with light depth and shinny bases**.

On the parietal part of the scalp appear two oval shaped areas without hair, confluated, with light depth and shinny bases (Figure 1).

Her mother mentioned that during the first trimester of the pregnancy (she did not know that she was pregnant) has taken Diclofenac amp. (five days) because she had abdominal pains. During pregnancy she did not have any infectious disease. Family history is negative.

**Figure 2:**
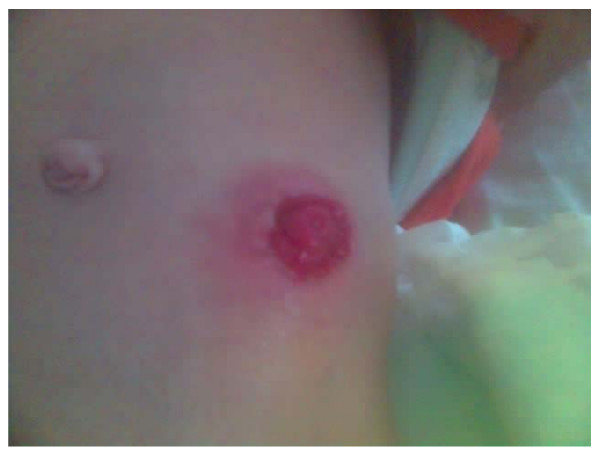
**Installed stoma (anus praeternaturalis)**.

In the surgery clinic the child was operated and treated under the diagnoses: Stenosis congenita ani and was installed stoma (Figure. 2). For associated defects in other organs, there are made specific consults.

**Figure 3:**
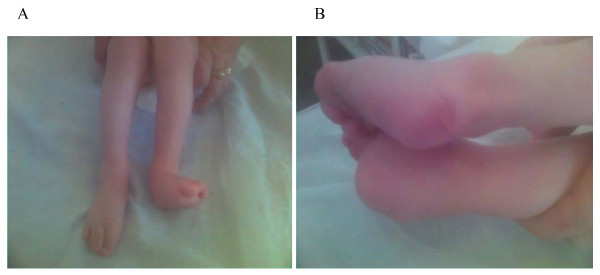
**a and b: Digiti V superductus pedis bill**. Laxitas articularum generalisata Sy Floppy.

Consultant orthopedist concluded the digiti V superductus pedis bilateralis, laxitas articularum generalisata and Sy Floppy (Figure 3a and Figure 3b).Consultant neurologist indicated minor visualmotor discoordination. Cardiologists and nephrolog consulting resulted on the common stage. Citogenetic report has shown Cariotype 46, XX with no changes on the chromosome number and structure.

The routine laboratory examinations resulted with common values except the blood SE rate, which was increased (60/1 h). After the antibiotic ordination (Cephalexin sir. A 125 mg), the same fall down to 15/1 h. The patient was discharged home with recommendation to follow up the pediatric surgeon visits.

## Conclusion

Aplasia cutis congenita is very rare anomaly (1:3000). Causes of congenital aplasia could be teratogenic substances [7]. Diclofenac is a non-steroidal anti-inflammatory drug (NSAID), commonly used by reproductive age women for the treatment of variety of conditions. NSAID inhibit the biosynthesis of prostanoids. Women may incidentally become pregnant while receiving NSAID therapy (unplanned pregnancy) [8]. The diclofenac crosses the placenta readily in the first trimester of human pregnancy resulting in a fetal diclofenac concentration. which is the same as the maternal serum concentration [9]. The drug may also accumulate in fetal tissue with time [9]. Usually diclofenac is given in multiple doses, so the possibility to reach teratogenic levels of fetal tissue concentrations is real in patients who are taking diclofenac. In our case the pregnant women received diclofenac 75 mg per day, five days. Diclofenac has also been shown to inhibit implantation and embryogenic development in rats when given on gestation day 5 [10]. A positive association between use of NSAID during pregnancy and miscarriages was reported by a study [11]. However, information regarding teratogenecity of NSAID during the critical period of organogenesis is lacking. Because aspirin and other NSAID share a similar mechanism of action (inhibition of prostaglandin synthesis) it was postulated that NSAID might induce congenital abnormalities when given during the critical period of organogenesis [12].

We present patient with aplasia cutis congenita accompanied by stenosis congenita ani, digiti V superductus pedis bill., laxitas articularum generalisata-sy Floppy and minor visualmotor discoordination. Based on mentioned studies and real clinical situation we concluded that Diclofenac used in early trimester of pregnancy might induce those abnormalities and considered reasonable to present this case.

## Abbreviations

NSAID: non-steroidal anti-inflammatory drug; Sy: Syndrome.

## Consent

Written informed consent was obtained from the patient's mother for publication of this case report and accompanying images. A copy of the written consent is available for review by the Editor-in-Chief of this journal.

## Competing interests

The authors declare that they have no competing interests.

## Authors' contributions

LP, SR, YSHM and MF analyzed and interpreted the patients' data. LP was a major contributor in writing the manuscript. All authors read and approved the final manuscript.
